# Co_3_V_2_O_8_ Nanoparticles Supported on Reduced Graphene Oxide for Efficient Lithium Storage

**DOI:** 10.3390/nano10040740

**Published:** 2020-04-13

**Authors:** Le Hu, Chaoqun Shang

**Affiliations:** 1 International Academy of Optoelectronics at Zhaoqing, South China Normal University, Zhaoqing 526060, China; hule@m.scnu.edu.cn; 2 Guangdong Provincial Key Laboratory of Optical Information Materials, South China Normal University, Guangzhou 510006, China

**Keywords:** rGO@CVO, anode materials, rate performance, cycling stability, lithium-ion batteries

## Abstract

Co_3_V_2_O_8_ (CVO) with high theoretical specific capacity derived from the multiple oxidation states of V and Co is regarded as a potential electrode material for lithium-ion batteries (LIBs). Herein, reduced graphene oxide (rGO)-supported ultrafine CVO (rGO@CVO) nanoparticles are successfully prepared via the hydrothermal and subsequent annealing processes. The CVO supported on 2D rGO nanosheets possess excellent structural compatibility for the accommodation of volume variation to maintain the structural integrity of an electrode during the repeated lithiation/delithiation process. On the other hand, the rGO, as a highly-conductive network in the rGO@CVO composite, facilitates rapid charge transfer to ensure fast reaction kinetics. Moreover, the CV kinetic analysis indicates that the capacity of rGO@CVO is mainly dominated by a pseudocapacitive process with favorable rate capability. As a result, the rGO@CVO composite exhibits improved specific capacity (1132 mAh g^−1^, 0.1 A g^−1^) and promising rate capability (482 mAh g^−1^, 10 A g^−1^).

## 1. Introduction

With the increasing environmental concerns and high demand for sustainable energy, there is great urgency to develop novel materials for high-performance energy storage and conversion [1,2,3]. Lithium-ion batteries (LIBs), as outstanding energy storage devices, have been widely used, owing to their high energy densities, long lifespans and environmental friendliness [4,5]. However, the traditional carbon-based anode shows limited specific capacity, which hinders the energy density of LIBs for further improvement [6,7,8]. Therefore, the investigation of high-efficiency anode materials for LIBs is highly essential for future applications [9,10,11].

Various types of anode materials, including carbonaceous, alloy reaction materials and transition metal oxides, have been widely investigated in field of LIBs [12,13,14]. Metal vanadate materials originated from multivalence of vanadium (V^5+^, V^4+^, V^3+^, V^2+^) have displayed high electrochemical kinetics [15,16]. Due to the bimetallic synergistic effect between the active constituents Co and V, cobalt vanadates have been regarded as considerable electrode materials in lithium storage [17]. Moreover, the advantages of the high theoretical specific capacity and low cost of metal vanadate have attracted more attention to it for perspective anode materials for LIBs [18,19]. Among them, Co_3_V_2_O_8_ (CVO) can absorb 15.4 Li^+^ during the first discharge process with higher lithium ion storage [19]. However, the unavoidable pulverization and the agglomeration of bulk CVO material during cycling for lithium storage result in poor electrochemical performance and cycle life [20]. To address these weak points, compositing CVO with carbon materials is a common tactic with which to enhance the conductivity and serve as volume buffer matrix for large volume change [21,22,23]. As a typical two-dimensional (2D) structure carbon material, reduced graphene oxide (rGO) has many advantages, including excellent structure compatibility, large special surface area and superior conductivity [24,25]. Moreover, rGO with a larger interlayer distance could well adapt to volume changes during the repeated cycling [26]. Therefore, incorporation of CVO with rGO is a promising way to fabricate advanced anode materials in lithium storage. 

In this work, CVO is composited with rGO (rGO@CVO), which is prepared via the hydrothermal and subsequent annealing processes. Ultrafine CVO nanoparticles attached onto the surface of rGO nanosheets can effectively mitigate the aggregation of nanomaterials and accommodate volume variation to maintain a highly-reversible capacity during repeated cycling. Meanwhile, the rGO nanosheets, as a conductive network, can enhance the conductivity of electrode, further facilitating fast charge transfer and ensuring favorable electrochemical properties at a high current density. Therefore, the rGO@CVO composite electrode exhibits efficient lithium storage with high reversible capacity and considerable rate capability. 

## 2. Experimental Section 

### 2.1. Materials

Graphene oxide (XFNANO, nanosheet diameter: 0.5–5 μm, thickness: 0.8–1.2 nm, Nanjing, China), sodium vanadate (Na_3_VO_4_, AR, Macklin, Shanghai, China), ammonia solution (NH_3_·H_2_O, 25%, Innochem, Beijing, China), cabaltous nitrate (Co(NO_3_)_2_·6H_2_O, 99%, Aladdin, Shanghai, China), poly(vinylidene fluoride) (PVDF, *M*_w_ = 534,000, Aldrich, Saint Louis, Missouri, MO, USA), N-methyl-2-pyrrolidone (NMP, Aldrich, Saint Louis, Missouri, MO, USA), super P (99.9%, Innochem, Beijing, China). All the chemicals were directly used without further purification.

### 2.2. Preparation of Samples 

Graphene oxide (GO) was directly purchased from XFNANO without further treatment [27]. The rGO@CVO composite was synthesized with a two-step method. In a typical process, 0.236 g Na_3_VO_4_ was dissolved into deionized water (DI, 30 mL) and heated up to 80 °C for 20 min to obtain a yellow solution. After cooling to room temperature, 3 mL NH_3_·H_2_O, 0.873 g Co(NO_3_)_2_·6H_2_O and 50 mg GO were immersed into the above solution under constant magnetic stirring for 30 min. The mixture was sealed for the hydrothermal process and maintained at 180 °C for 12 h. After cooling down, the sediment was thoroughly washed with absolute ethanol and DI three times followed by freeze-drying for 24 h. Next, the dry sediment was annealed at 400 °C for 3 h in Ar atmosphere to obtain rGO@CVO composite. In addition, the CVO nanoparticles were prepared with a similar treatment process without the addition of GO.

### 2.3. Material Characterization 

X-ray diffraction (XRD, Bruker D8 advance, Cu *K*α radiation (*λ* = 1.5406 Å; voltage: 40 kV; current: 40 mA, Karlsruhe, Germany) was introduced to confirm the crystal structure of rGO@CVO. The chemical composition of rGO@CVO was investigated by X-ray photoelectron spectroscopy (XPS, PHI 5600, Physical electronics, Chanhassen, MN, USA). The typical morphologies of the all the samples were characterized by field-emission scanning electron microscopy (FESEM, voltage: 2 kV, ZEISS Ultra 55, Carl Zeiss Inc., Oberkochen, Germany) and transmission electron microscopy (TEM, a JEOL 2010F, JEOL Ltd., Tokyo, Japan). The EDX mappings were obtained by FESEM with the voltage of 10 kV.

### 2.4. Electrochemical Measurements

In total, 2016 coin-type cells were assembled in an argon-filled glovebox (MIKROUNA, Universal (1800/750/900), Shanghai, China) for electrochemical tests. A Li disk with diameter of 15 mm was used as the counter electrode; 1 M LiPF_6_ dissolved in EC/DMC, (volume ratio of 1/1) was the electrolyte. The slurry containing rGO@CVO (70 wt %), Super P (20 wt %) and PVDF (10 wt %) was homogenously dispersed into NMP solution and then coated on Cu foil, which was dried at 60 °C for 24 h as the working electrode. The charge/discharge tests were performed by the Neware battery testing system in the voltage between 0.01 and 3.0 V. Cyclic voltammetry (CV) and electrochemical impedance spectroscopy (EIS) were measured on the CHI 660E electrochemical workstation (Chenhua Inc., Shanghai, China).

## 3. Results and Discussion

**Figure 1:**
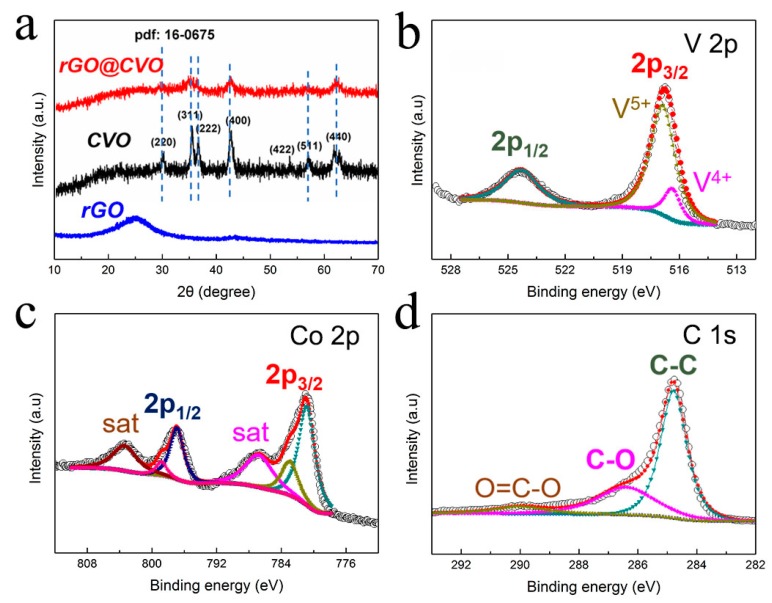
(**a**) XRD patterns of the CVO, rGO@CVO and rGO. XPS spectra of (**b**) V, (**c**) Co and (**d**) C of the rGO@CVO composite, respectively.

X-ray diffraction was performed to characterize the structure and crystallization of the CVO nanoparticles and rGO@CVO composite. As depicted in Figure 1a**,** the diffraction peaks of the CVO nanoparticles appear at 18.8°, 35.9°, 43.3°, 57.7° and 62.0°, which match well with the (120), (221), (122), (042), (162) and (442) facets of CVO (JCPDS number 16-0675). The XRD peaks of rGO@CVO composite are similar to those of CVO nanoparticles but with weaker intensity and broader shape, indicating that the addition of rGO could decrease the crystal size of CVO nanoparticles [28,29]. The chemical oxidation of the samples was investigated by XPS measurement. In high-resolution XPS of V 2p spectra (Figure 1b) [30], the major signal of V 2p_3/2_ two peaks centered at 517.5 and 516.7 eV are ascribed to the V^5+^ and V^4+^. The larger peak area of V^5+^ indicates the majority of V^5+^ in the rGO@CVO composite. In Figure 1c, the detailed Co 2p spectrum possesses two spin-orbit doublets that are characteristic of Co^2+^ and Co^3+^. In addition, two characteristic satellites at 803.1 and 786.7 eV indicate the high-spin Co^2+^ of the rGO@CVO composite [31]. These results confirm that the mixture of V^5+^, V^4+^, Co^2+^ and Co^3+^ in the rGO@CVO composite. The C 1s spectrum is divided into three peaks at 289.8, 286.8 and 284.9 eV, corresponding to the C=O (or O–C=O), C–O and C=C bonds, respectively (Figure 1d) [32].

**Figure 2:**
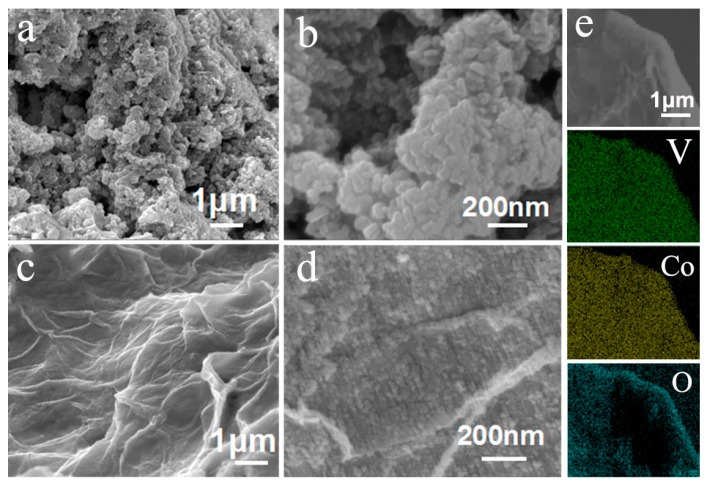
FESEM images of (**a**,**b**) CVO nanoparticles and (**c**,**d**) rGO@CVO composite. (**e**) EDX mapping images of V, Co and O in the rGO@CVO composite.

Typical morphologies of CVO nanoparticles and rGO@CVO composite were investigated via SEM and TEM. Figure 2a,b shows that the pure CVO nanoparticles are spontaneously agglomerated with the uniform size of about 60–80 nm. The SEM morphologies of rGO@CVO composites are displayed in Figure 2c,d. The rGO nanosheets act as a support skeleton with obvious wrinkles. Furthermore, the high-resolution SEM image shows that ultrafine CVO nanoparticles uniformly cover the surfaces of rGO nanosheets with an average size of about 10–20 nm. The average size of CVO nanoparticles in the rGO@CVO composite is much smaller than that of the pure CVO, which is in accordance with the XRD results. The original SEM image and corresponding EDX mappings reveal the uniform distribution of Co, V and O elements on the surface of rGO in rGO@CVO composite (Figure 2e).

**Figure 3:**
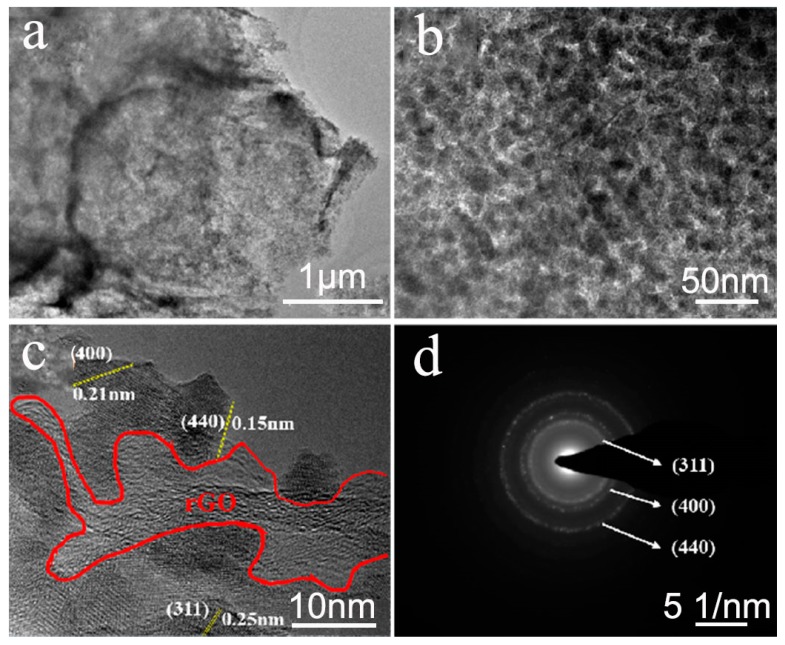
(**a**) The low-resolution and (**b**,**c**) high-resolution TEM images of rGO@CVO composite; (**d**) the SAED pattern of rGO@CVO composite.

The TEM image shows that numerous ultrafine CVO nanoparticles are grown on the surfaces of rGO nanosheets (Figure 3a). From the further magnified view of rGO@CVO composite in Figure 3b, the CVO nanoparticles have a diameter range from 10 to 20 nm. These results agree well with the FESEM analysis. Furthermore, in the HRTEM image (Figure 3c), the extinct inter–planar distances of 0.15, 0.21 and 0.25 nm correspond to the (440), (400) and (311) planes of the CVO phase, respectively. The selected area electron diffraction (SAED, Figure 3d) exhibits a multi-ring shape with a polycrystalline character, where the diffraction rings are indexed to the CVO crystal planes.

**Figure 4:**
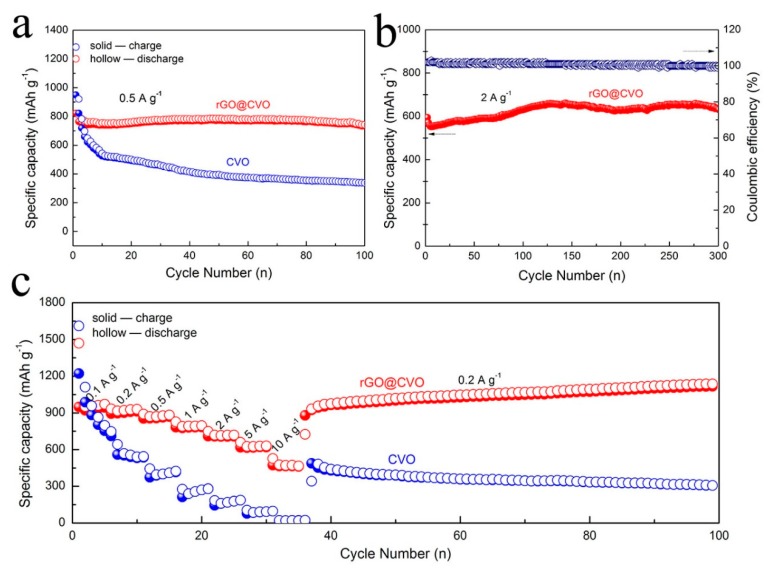
(**a**) Cycling performance of rGO@CVO and CVO at 0.5 A g^−1^; (**b**) long-term cycling performance of rGO@CVO electrode at 2 A g^−1^; (**c**) rate capability of rGO@CVO and CVO electrodes.

The morphological and structural characterizations suggest that the hierarchical rGO@CVO composite is prepared successfully via a facile two-step method. Inspired by the combination of the ultrafine structure of CVO nanoparticles and the high conductivity of rGO, the electrochemical property of the rGO@CVO for lithium storage was investigated. Figure 4a exhibits the cycling performance of rGO@CVO and CVO electrodes at the current density of 0.5 A g^−1^. The rGO@CVO electrode delivers the specific capacity of 738 mAh g^−1^ with no obvious decay after 100 cycles. In contrast, the specific capacity of CVO electrode sharply decreases at the first 10 cycles and gradually reduces to only 340 mAh g^−1^ at the end of 100 cycles. Additionally, after 300 cycles, the rGO@CVO electrode keeps a high reversible capacity of 633 mAh g^−1^ at high rate of 2 A g^−1^ (Figure 4b). The favorable cycling performance suggests that the incorporation of CVO nanoparticles with rGO can accommodate volume variations to ensure high reversible capacity and cycling stability. Figure 4c displays the rate capability of rGO@CVO and CVO electrodes. As the current density increases from 0.1 to 10 A g^−1^, the specific capacity of rGO@CVO electrode decreases from 965 to 482 mAh g^−1^. Nearly 50% capacity retention is achieved at a high rate, which reveals the superior rate performance of the rGO@CVO electrode. Meanwhile, after 100 cycles, the specific capacity gradually increases up to 1132 mAh g^−1^ at 0.2 A g^−1^, which could be ascribed to the full activation process at a high rate. In contrast, the CVO electrode shows a negligible capacity at a high rate (14 mAh g^−1^, 10 A g^−1^). As shown in Table 1, the rGO@CVO electrode in lithium storage exhibits considerable electrochemical performance. The promising cycling and rate performance of the rGO@CVO electrode can be attributed to 2D rGO nanosheets possessing excellent structure compatibility to protect the structural integrity of rGO@CVO electrode, thereby maintaining long-term cycling stability during charging and discharging processes. Moreover, the rGO, as a highly-conductive network in the rGO@CVO composite facilitates fast electron and ion transport to ensure a promising rate capability.

**Figure 5:**
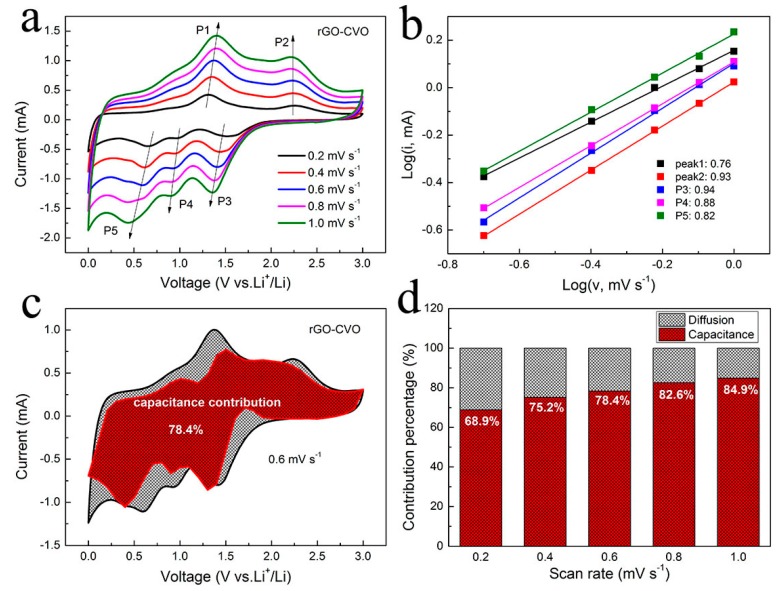
Kinetic analysis for lithium storage of rGO@CVO electrode: (**a**) CV curves at various scan rates; (**b**) log(*i*) vs. log(*v*) plots at each redox peaks. (**c**) Capacitive and diffusion-controlled contributions of rGO@CVO electrode at scan rate of 0.6 mV s^−1^; (**d**) pseudocapacitive contribution ratio at different sweep rates of rGO@CVO electrode.

To further illustrate the favorable rate performance of rGO@CVO electrode, the reaction kinetics of the rGO@CVO electrode were investigated by CV measurement. As illustrated in Figure 5a, the CV curves of rGO@CVO electrode show almost the same trend shape with an increased sweep rate. Generally, the linear relation between *i* (current density: mA) and *v* (scan rate: mV s^−1^) can be assigned as follows, by the equation of *i* = *av^b^*, where *a* and *b* are constants [25,33]. The slope of the fitted line (log (*i*) = log(*a*) + *b*log(*v*)) determines the *b* value, which is applied to analyze the capacitive process of the electrode. If the *b* value is close to 1, the surface pseudocapacitance-controlled process (interfacial Li^+^ storage) is dominant. The *b* value approaching to 0.5 represents a dominant diffusion-controlled electrochemical process [24,34]. Based on anodic and cathodic peaks of rGO@CVO electrode, in Figure 5b, the *b* values are calculated to be 0.76, 0.93, 0.94, 0.88 and 0.82, respectively. The high *b* values demonstrate a behavior controlled by pseudocapacitance, further implying fast Na^+^ transport. The surface pseudocapacitive portion of rGO@CVO electrode can be performed quantitative calculation according to the equation of *i* = *k*_1_*υ*^1/2^ + *k*_2_*υ* × (*i/υ*^1/2^ = *k*_1_ + *k*_2_*υ*^1/2^) [24,35]. Typically, at 0.6 mV s^−1^ (Figure 5c)**,** ≈78.4% of the total capacity in the rGO@CVO electrode comes from the surface’s capacitive contribution. Moreover, Figure 5d shows that the surface’s pseudocapacitive contribution in the rGO@CVO electrode is obviously improved from 68.9% (0.2 mV s^−1^) to 84.9% (1.0 mV s^−1^). The high ratio of pseudocapacitance contribution can effectively enhance the reversible capacity and rate capability at a high current density for lithium storage.

**Figure 6:**
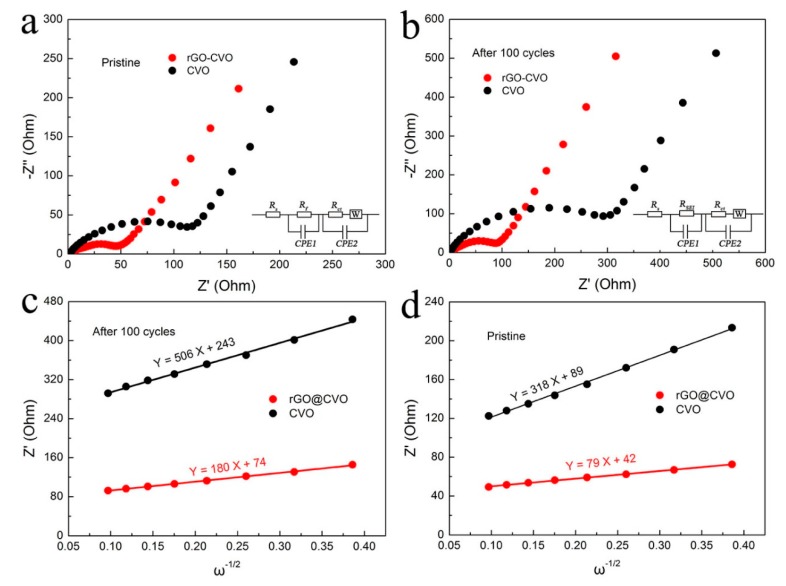
Nyquist plots of rGO@CVO and CVO electrodes: (**a**) pristine and (**b**) after 100 cycles; the relationship plot of the low-frequency region between *Z*’ and *ω*^−1/2^: (**c**) pristine and (**d**) after 100 cycles.

The EIS measurement was applied to evaluate the reaction kinetics of rGO@CVO composite at the electrode/electrolyte interface for LIBs. In the EIS profile, the semicircle in the high frequency region represents the charge-transfer resistance (*R*_ct_), implying the resistance on the electrode-electrolyte interface [36,37]. As shown in Figure 6a, the *R_ct_* values of rGO@CVO and CVO electrodes in pristine state are about 55 and 144 Ω. After 100 charge/discharge cycles, the *R*_ct_ of rGO@CVO and CVO electrodes increase to 102 Ω and 334 Ω, which could be attributed to the formation of stable SEI film and structural stability (Figure 6b) [38]. The smaller *R*_ct_ values of pristine and cycled rGO@CVO electrode demonstrate that the rGO@CVO electrode possesses stable structure and good electrical conductivity, revealing the fast electron transfer and reaction kinetics of rGO@CVO electrode. The Na^+^ diffusion coefficient (*D*_Na+_) is calculated by the equation of *D* = *R*^2^*T*^2^/(*2n*^4^*A*^2^*F*^4^*C*^2^*b*^2^) [39,40]. The *b* is the Warburg factor, which is the slope of the line *Z’* = *R*_e_ + *R*_ct_ + *bω*^−1/2^ [41]. In the analysis of the equations, the *b* value dominates *D*_Na+_; the smaller the b value, the larger the *D*_Na+_. Obviously, the *b* value of the rGO@CVO electrode is much lower than for the CVO electrode, which demonstrates that the rGO@CVO electrode possesses the larger *D*_Na+_ and fast diffusion kinetics to guarantee favorable electrochemical performance at high rate for efficient lithium storage (Figure 6c,d).

## 4. Conclusions

In summary, we successfully synthesized the hierarchical rGO@CVO composite for lithium storage. The CVO supported on 2D rGO nanosheets possess excellent structural compatibility for accommodation of volumetric variation to maintain structural integrity of electrode during the repeated lithiation/delithiation process. On the other hand, the rGO, as the highly-conductive network in the rGO@CVO composite, facilitates rapid charge transfer to ensure promising rate capability. As expected, the rGO@CVO composite for lithium storage exhibits a highly-reversible capacity (1132 mAh g^−1^, 0.1 A g^−1^) and considerable rate performance (482 mAh g^−1^, 10 A g^−1^).

## Figures and Tables

**Table 1:** A comparison of the electrochemical performances of rGO@CVO (this work) and other attempts in lithium ion batteries.

Samples	Lithium Ion Batteries	
	Cycling Performance (Reversible Capacity)	Rate Performance
rGO@CVO (This work)	738 mAh g^−1^ at 0.5 A g^−1^	482 mAh g^−1^ at 10 A g^−1^
rGO@Co_3_V_2_O_8_ NP (Ref. [17])	1050 mAh g^−1^ at 0.05 A g^−1^	161 mAh g^−1^ at 10 A g^−1^, 120 mAh g^−1^ at 20 A g^−1^
C–CVO/400 (Ref. [18])	735 mAh g^−1^ at 1 A g^−1^	422 mAh g^−1^ at 10 A g^−1^
Co_3_V_2_O_8_ HMMSs (Ref. [20])	984 mAh g^−1^ at 0.5 A g^−1^	545 mAh g^−1^ at 2 A g^−1^
